# Regulatory T Cells, a Viable Target Against Airway Allergic Inflammatory Responses in Asthma

**DOI:** 10.3389/fimmu.2022.902318

**Published:** 2022-06-10

**Authors:** Jing Zhang, Yuan Zou, Longmin Chen, Qianqian Xu, Yi Wang, Min Xie, Xiansheng Liu, Jianping Zhao, Cong-Yi Wang

**Affiliations:** ^1^ Department of Respiratory and Critical Care Medicine, The Center for Biomedical Research, NHC Key Laboratory of Respiratory Disease, Tongji Hospital Research Building, Tongji Hospital, Tongji Medical College, Huazhong University of Science and Technology, Wuhan, China; ^2^ Department of Rheumatology and Immunology, The Central Hospital of Wuhan, Tongji Medical College, Huazhong University of Science and Technology, Wuhan, China; ^3^ Department of Respiratory and Critical Care Medicine, Shanxi Bethune Hospital, Shanxi Academy of Medical Sciences, Tongji Shanxi Hospital, Third Hospital of Shanxi Medical University, Taiyuan, China

**Keywords:** regulatory T cells, allergic airway inflammation, asthma, airway epithelial repair, therapeutic strategies

## Abstract

Asthma is a multifactorial disorder characterized by the airway chronic inflammation, hyper-responsiveness (AHR), remodeling, and reversible obstruction. Although asthma is known as a heterogeneous group of diseases with various clinical manifestations, recent studies suggest that more than half of the clinical cases are ‘‘T helper type 2 (Th2)-high’’ type, whose pathogenesis is driven by Th2 responses to an inhaled allergen from the environmental exposures. The intensity and duration of inflammatory responses to inhaled allergens largely depend on the balance between effector and regulatory cells, but many questions regarding the mechanisms by which the relative magnitudes of these opposing forces are remained unanswered. Regulatory T cells (Tregs), which comprise diverse subtypes with suppressive function, have long been attracted extensive attention owing to their capability to limit the development and progression of allergic diseases. In this review we seek to update the recent advances that support an essential role for Tregs in the induction of allergen tolerance and attenuation of asthma progression once allergic airway inflammation established. We also discuss the current concepts about Treg induction and Treg-expressed mediators relevant to controlling asthma, and the therapies designed based on these novel insights against asthma in clinical settings.

## Introduction

Asthma is a chronic airway inflammatory disease that affects more than 350 million individuals worldwide (1). The etiology underlying asthma includes both genetic predisposition and environmental exposures (2). In general, the airway inflammation in asthmatic setting arises from the reaction in response to allergens and/or other environmental factors, thereby leading to an aberrant airway Th2-type immune response (3). Although a great effort of studies had advanced the understanding of pathologic features of asthma, its mechanisms underlying the regulation of allergic airway inflammation are yet to be fully elucidated. As a result, the development of novel therapeutic strategies against asthma is confronted with formidable challenges.

There is strong evidence in animals that regulatory T cells (Tregs) act as a key regulator of allergic diseases and are essential to limit antigen-specific immune responses. For example, mice deficient in CD4^+^CD25^+^ Tregs resulted from loss-of-function mutations in the *Foxp3* gene are featured by the development of spontaneous autoimmunity, lymphoproliferation, allergic airway inflammation, hyper IgE syndrome, and eosinophilia (4). Similarly, adoptive transfer of ovalbumin (OVA) peptide-specific CD4^+^CD25^+^ Tregs into the OVA-sensitized mice attenuated airway hyper-responsiveness (AHR) along with reduced number of eosinophils and production of Th2 cytokines in the lung following allergen challenge (5). Foxp3^+^ Tregs also suppress chronic allergic inflammation to establish allergen-tolerance in the respiratory mucosa (6). Furthermore, manipulation of steroid responsiveness in Tregs could represent a novel strategy to treat steroid refractory asthma, as their responsiveness determines steroid sensitivity during allergic airway inflammation (7). Collectively, these studies underscore the significance of Tregs in the regulation of allergic airway inflammation in mouse models.

Unlike the impact observed in animal models, the role of Tregs in asthmatic patients is yet to be well defined. Studies revealed that adult asthmatic patients with either stable or exacerbated symptoms displayed lower percentage of Tregs along with impaired suppressive function in the blood and airway (8). Similarly, decreased pulmonary Treg number coupled with lower capability to suppress pulmonary Th2 responses were observed in asthmatic children (9). In sharp contrast, some studies also demonstrated that the amount of airway Tregs was increased in adult patients with moderate to severe asthma as compared to both mild asthmatic patients and healthy subjects (10), especially in response to bronchial allergen provocation (11). The discrepancy between these findings could be caused by the differences of study cohorts and the approaches for Treg analysis. Nevertheless, a consistent conclusion could probably be reached for the impaired Treg function in asthmatic patients, particularly for their capability to suppress Th2 responses. A recent study further suggested that the numerical and functional defects of Tregs may render the children and younger adults more susceptible to asthma, while the relationship between Tregs and asthma risk or severity in older patients is relatively weak (12). Although the contribution of Tregs in asthma is not fully addressed, clinical improvement following allergen immunotherapy (AIT) for asthma suggested an association with the induction of IL-10-, IL-35- and TGF-β-producing Tregs and Foxp3^+^ Tregs (13). Therefore, in this review we seek to summarize the immunological features of allergic asthma, and then update the recent advances that support the role of Tregs in allergen tolerance induction and in limiting disease severity once allergic airway inflammation established. We also discuss the current concepts about Treg induction and Treg-expressed mediators relevant to controlling asthma, and the therapies designed based on these novel insights against asthma in clinical settings.

## The Immunological Characteristics Underlying Allergic Asthma

Type 2 immunity has now been well recognized to be a critical feature relevant to a complex network of immunologic mechanisms in allergic asthma (14). Type 2 immune response involves an ever-expanding repertoire of immune cells, including Th2 cells, B cells, natural killer (NK) cells, NKT cells, basophils, eosinophils, mast cells, and group 2 innate lymphoid cells (ILC2s) and their associated cytokines (15). IL-4, IL-5, IL-9, and IL-13 are predominantly produced by immunocytes, while IL-25, IL-31, IL-33, and thymic stromal lymphopoietin (TSLP) are released from tissue cells, particularly epithelial cells (16).

The immunological mechanisms underlying allergic response can be classified into two main phases: (1) the sensitization and memory phase, and (2) the effector phase. The later can be subdivided into the immediate and late-phase reactions (17, 18). During the sensitization and memory phase, the differentiated and clonal expanded allergen-specific Th2 cells produce copious amount of IL-4 and IL-13 to drive the class switching of antibody isotypes to the ε heavy chain. The allergen-specific IgE then binds to the high-affinity FcϵRI on the surface of mast cells and basophils, thereby contributing to the IgE sensitization of individuals against allergens. In this phase, a memory pool of allergen-specific Th2 and B cells is also generated, which is ready to act upon allergen encounters. The immediate reaction of allergic response is also termed as type 1 hypersensitive reaction. Upon the challenge from same allergens, crosslinking of the IgE-FcϵRI complexes on the sensitized basophils and mast cells leads to the release of anaphylactogenic mediators (such as vasoactive amines, prostaglandin D, platelet-activating factor, leukotriene, chemokines, and other cytokines) responsible for the classical immediate symptoms of allergic disease. The late-stage reaction generally occurs following 4-6 hours of allergen stimulation and lasts for more than a few days, and is featured by the localized inflammatory responses mediated by the infiltrated eosinophils, neutrophils, macrophages, Th2 cells and basophils. The ongoing inflammation results in more severe clinical manifestations of allergy, such as chronic persistent asthma, allergic rhinitis, and in extreme cases, systemic anaphylactic reactions (18).

Recent studies also suggested the involvement of epithelial cells in allergic pathology. Barrier epithelial cells not only represent the very first line of defense against environmental insults, but also produce cytokines (e.g., IL-25, IL-31, IL-33, and TSLP) and alarmins (e.g., uric acid, ATP, HMGB1, and S100 proteins) following allergen exposures (19). These signals constitute important factors in the early phase of asthma and promote Th2 differentiation through their effect on tissue dendritic cells and ILC2s (20). In particular, there is evidence that a neutralizing mAb against IL-25 results in a significantly reduced production of IL-5 and IL-13 along with attenuated eosinophil infiltration, goblet cell hyperplasia, and serum IgE secretion, by which it prevents AHR following OVA-induced allergic airway inflammation in mice (21). More excitingly, blocking antibodies against either TSLP or IL-33/ST2 signaling are currently at different stages of clinical trials, which could be promising candidates for asthma treatment in clinical settings (22, 23).

ILCs are defined as ILC type1, 2, and 3 cells based on their transcription factors and cytokine production patterns, and among which, ILC2s play a substantial role in the initiation, progression, and steroid resistance of allergic airway inflammation (20). It was noted that IL-33 targets ILC2 to produce IL-5 and IL-13, thereby enhancing eosinophil recruitment, goblet cell hyperplasia, macrophage M2 polarization, dendritic cell (DC) activation and Th2 differentiation (24–27). Studies revealed that the number of total and type 2 cytokine-expressing ILC2s is significantly higher in the peripheral blood and airway of patients with systemic steroid-dependent severe eosinophilic asthma than those of patients with mild asthma (28). Given that the intracellular cytokine expression by Th2 cells within the airways did not differ between the above two groups of patients, The observations support that uncontrolled ILC2s rather than Th2 cells, represent a steroid-insensitive population of cells to exacerbate the development of airway inflammation in patients with severe prednisone-dependent eosinophilic asthma (28). Other distinct types of effector T cells (Teffs) may also get involved in continuous allergic inflammation as well. For example, although Th1 and IFN-γ secreting NKT cells induce epithelial apoptosis through cell-mediated cytotoxicity, they also exert an inhibitory role in Th2 cells and suppress IgE isotype switching (29). While IL-17 producing Th17 cells mediate neutrophilic type inflammation other than exacerbating Th2-related allergic inflammation (30). Moreover, Th9 cells employ multiple mechanisms to orchestrate allergic inflammation, and particularly, their interaction with diverse cell types including mast cells, ILCs, and DCs, to promote coordinated regulation of allergic airway inflammation (31, 32). Other than secretion of their signature cytokine IL-9, Th9 cells from mice and humans also secret other cytokines such as IL-10, IL-17, IL-21, and IL-22, to facilitate immune responses in the setting of allergic asthma (33, 34). Furthermore, studies on atopic dermatitis demonstrated feasible evidence supporting that the expansion of Th2/Tc2 and Th22/Tc22 may also exert an important role in allergic inflammation (14, 35).

## Origins and Subtypes of Tregs

Tregs are one of the main bastions against inappropriate or over-exuberant inflammatory responses, and play an indispensable role in the maintenance of immune tolerance in asthma (36). However, subsets of CD8^+^ T cells, CD4^-^CD8^-^ T cells, γδ T cells, regulatory B cells (Bregs), IL-10-producing DCs, IL-10-producing NK cells, and macrophage subsets with suppressive properties also contribute to the suppressive and regulatory events (37). Currently, two main subsets of Tregs have been defined: the thymus-derived naturally occurring CD4^+^CD25^hi^ Foxp3^+^ Tregs, also called tTregs, and the peripherally induced adaptive Tregs (pTregs) (38, 39). pTregs are further divided into Foxp3^+^ pTregs, Foxp3^−^ IL-10-producing Tr1 cells, and Foxp3^−^ TGF-β-expressing Th3 cells. Studies in animals suggest the Foxp3^+^ pTregs and IL-10-producing Tr1 cells may contribute to the differences of asthma susceptibility associated with different genetic background (40). Generally, Foxp3 induction in tTregs can occur at the double positive (DP) stage or preferentially at the CD4 single positive (SP) stage or during the transition to this stage in the thymus (41). Interaction with antigen presented by either cortical or medullary thymic epithelial cells is sufficient to induce Foxp3 expression, thereby committing T-cell precursor to Treg lineage (42). pTregs are differentiated in the secondary lymphoid organs and tissues, and they are particularly enriched in the intestinal mucosa and lung during chronic inflammation, with specificities directed against food antigens, gut microflora, and environmental allergens (43). The induction of pTregs at the gastrointestinal tract is facilitated by the CD103^+^CD11c^+^ DCs in a TGF-β and retinoic acid-dependent manner (44–46), while lung tissue-resident macrophages constitutively coexpressing TGF-β and retinal dehydrogenases (RALDH1 and RALDH2) are the main subset of cells driving pTreg generation from naïve CD4^+^ T cells (47). It is worthy of note that the classification of Tregs could vary based on the specific markers employed. For example, Tregs can be also classified into nTregs, iTregs, ICOS^+^ Tregs, Tr1, CD8^+^ Tregs and IL-17-producing Tregs (48); however, some of these Treg subsets could be functionally overlapping or synergizing each other.

tTregs and pTregs are phenotypically distinct, and display different specificities and complementary functions *in vivo* (49). Generally, TCR on tTregs primarily recognizes self-antigens, which is crucial for establishing self-tolerance and preventing autoimmunity, while pTregs are thought to predominantly govern tolerogenic responses against non-self-antigens, such as allergens, food, and the commensal microbiota (50, 51). In a mouse model of chronic asthma, passive transfer of pTregs efficiently suppressed all aspects of asthmatic phenotype, whereas equal amount of tTregs only manifested a modest impact in this model, indicating that pTreg are substantially more tolerogenic in this setting (52). Although both tTregs and pTregs attenuate the development of asthma in WT recipients, those cells, however, enhance lung allergic responses in CD8^-/-^ recipients (53). The reprogramming pathways and enhancement appear to be distinct and cytokine specific, in which IL-13 production in nTreg depends on the GITR signaling, while IL-17 production in pTregs is induced by IL-6 signaling (53). There is evidence that tTregs stability in allergic settings is maintained by the epigenetic mechanisms and metabolites generated by themselves such as cyclic adenosine monophosphate (cAMP) (50). In contrast, the instability of Foxp3 expression and loss of suppressive function in pTreg are closely related to the methylation state of the Treg-specific demethylated region (TSDR) (50). Therefore, the regulatory mechanisms underlying the maintenance of Treg stability and functionality are essential to the development of effective strategies against allergic airway diseases.

## Cellular and Molecular Mechanisms Underlying Treg Attenuation of Asthma

**Figure 1:**
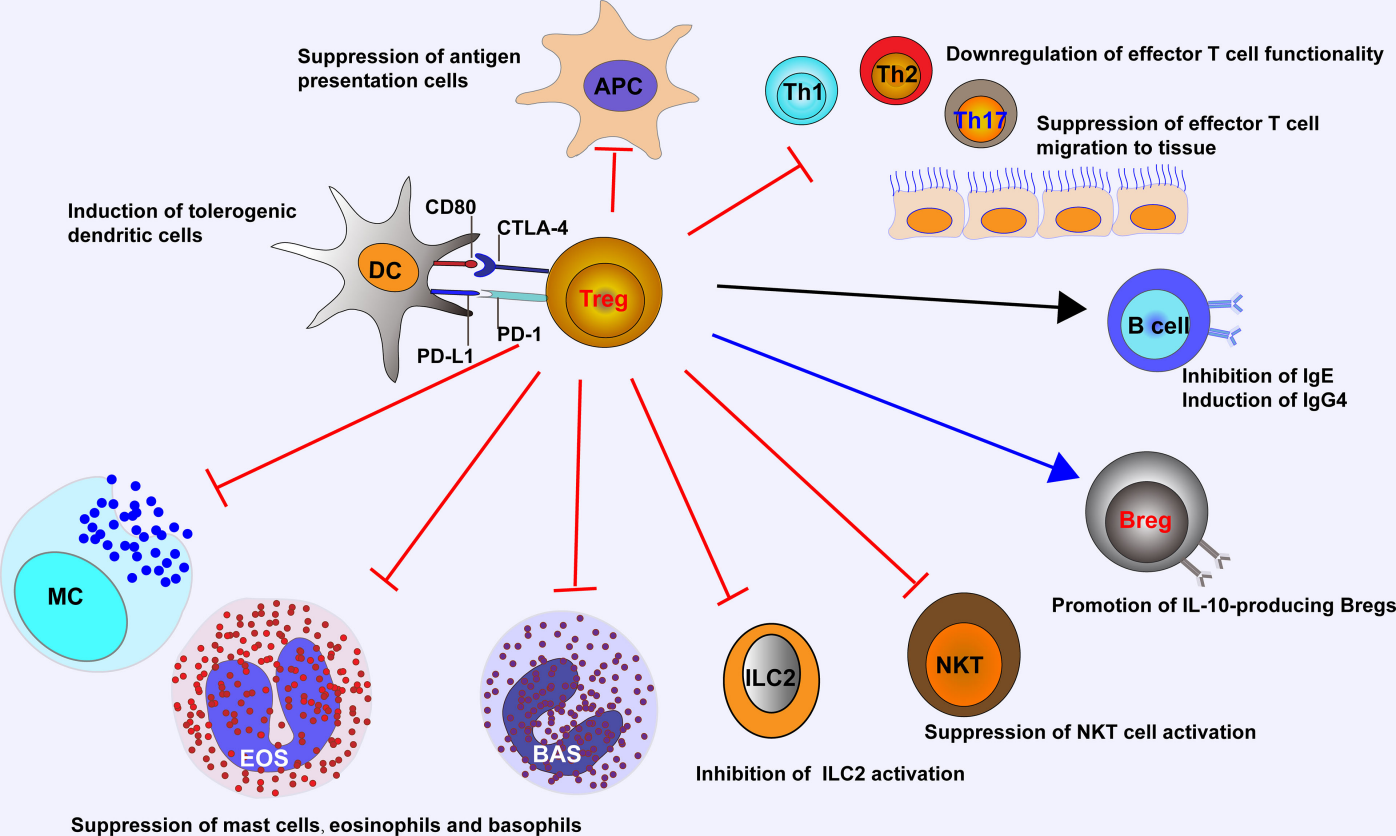
Tregs control ongoing inflammation by acting on major cells that drive allergic reaction, including antigen presentation cells, Teffs, NKT cells, ILC2s, eosinophils, basophils, and mast cells. Tregs suppress IgE-producing B cells, while induce IgG4-producing B cells and IL-10-producing Bregs, and promote the generation of tolerogenic dendritic cells. APC, antigen presentation cell; EOS, eosinophil; BAS, basophil; MC, mast cell.

The suppressive functions of Tregs in allergic inflammation are mediated by an ever-growing list of mechanisms (**Figure 1**). Tregs can suppress antigen presentation cells (APCs) to activate Teffs, while enhancing the function of tolerogenic DCs. Tregs inhibit the migration and functionality of Teffs including Th1, Th2, and Th17 cells. Tregs also repress the secretion of allergen-specific IgE from plasma cells and induce IgG4-secreting B cells and IL-10-producing Bregs. Other than Teffs, Tregs are able to suppress the activation of eosinophils, basophils, mast cells, NKT cells, and ILC2s (14, 54). The above-mentioned suppressive functions from Tregs are introduced by a number of soluble and membrane-bound molecules such as cytokines with inhibitory effect (e.g., IL-10, TGF-β and IL-35), enzymes or proteins relevant to cytolysis (e.g., granzymes A, B and K, and perforin), membrane proteins and signaling molecules relevant to metabolic homeostasis (e.g., CD25, CD39, CD73, cAMP, LAG3, adenosine receptor 2, and histamine receptor 2), and surface molecules (e.g., PD-1 and CTLA-4) for targeting DCs (38, 55–57).

The key role played by IL-10 and TGF-β in the context of allergic asthma is now well established (summarized in **Table 1**). Apart from Tregs, IL-10 is also released by Bregs, monocytes, a small fraction of NK cells, macrophages, DCs and ILCs (15). IL-10 exerts its effect on both innate and adaptive immune responses, thereby inducing immune tolerance and dampening tissue inflammation (90). For example, transfer of OVA peptide-specific Tregs to OVA-sensitized mice repressed AHR, eosinophil recruitment, and Th2 cytokine expression in the lung following allergen challenge (5), which was reversed by the application of IL-10R blocking antibody (5). It is worthy of note that the IL-10-producing Tr1 cells also represent an essential mechanism in immune tolerance to a high dose of allergens in nonallergic individuals, such as high dose bee venom exposure in beekeepers by natural bee stings (55).

**Table 1:** Summarized functions of IL-10 and TGF-β in allergic asthma.

IL-10	TGF-β
Inhibits antigen present cell (APC) maturation, antigen presentation and pro-inflammatory cytokine secretion (58) Induces IL-10-producing DCs (59)	Inhibits DC maturation and antigen presentation; promotes Langerhans cell development (60, 61) Stimulates cells at the resting state (monocytes), whereas activated cells (macrophages) are inhibited (62)
Inhibits mast cell activation and release of pro-inflammatory cytokines (63) Inhibits eosinophil and basophil cytokine production (5) Suppresses ILC2 activation and cytokine production (64)	Inhibits expression of FcϵRI (65) Promotes chemotaxis of neutrophils, eosinophils, and mast cells (66–68)
Suppresses allergen-specific Teffs (69)	Suppresses allergen-specific Teffs (70, 71) Associates with CTLA-4 expression on T cells (72) Promotes T cell survival (73)
Suppresses IgE (74) Induces IgG4 and IgA (75)	Suppresses class switching to the majority of IgG isotypes (76, 77) Suppresses IgE (78) Induces IgA (79)
Enhances B cell survival (75, 80)	Inhibits B cell proliferation (81) Promotes apoptosis of naïve or immature B cells (82, 83)
Promotes the generation of Tr1 cells (84)	Induces Foxp3 and suppressive function of Tregs (72, 85) Induces Th9, Th17 and Tfh cells under different conditions (86–88)
Synergistic effect in *in vivo* suppression with CTLA-4, PD-1 and TGF-β (89)	Synergistic effect in *in vivo* suppression with CTLA-4, PD-1 and IL-10 (89)

TGF-β is a pleiotropic cytokine and Tregs are the major source of its secretion. The implication of TGF-β in allergic asthma is complicated, and confronting effects are observed (38). It has been recognized that TGF-β produced by Tregs is indispensable for the *in vivo* pTreg expansion and immunomodulatory functionality in an autocrine manner, which is crucial for the induction of immune tolerance and design of alternative mucosal vaccination strategies. However, TGF-β has also been noted to increase mucus production, promote airway remodeling and fibrosis in asthmatic settings, which could be prevented by the anti-TGF-β therapy in allergen challenged animals (91). Therefore, further studies are required to elucidate the paradoxical role of TGF-β in the regulation of allergic asthma.

## The Implication of Tregs in Airway Remodeling

Airway remodeling refers to the pathological restructuring of the small and large airways in asthma, including neovascularization, subepithelial fibrosis, loss of epithelial integrity, goblet cell and mucus gland enlargement, and increased airway smooth muscle (ASM) mass (92). These pathophysiological changes result in alterations in the composition and structural organization of molecular and cellular components that constitute the airway. As a consequence, the asthmatic patients manifest the presence of airway narrowing and edema, AHR, and mucus hypersecretion, which is relevant to the poor clinical outcomes in asthmatic patients (93).

Airway remodeling is induced by factors from both inflammatory cells and structural cells. In general, the change of structural cells is under the influence of inflammatory cells (94). A variety of inflammatory cells presented in the asthmatic airways is able to produce mediators that have the potential to induce airway remodeling, such as cysteinyl leukotrienes (CysLTs), IL-13, endothelins, TGF-β, and epidermal growth factor (EGF). Vascular endothelial growth factor (VEGF) is an angiogenic factor, which has also been shown to induce airway remodeling and enhance Th2-mediated lung inflammation (95). Secretion of VEGF by cultured ASM cells is upregulated in response to the proinflammatory cytokines, TNF-α and IL-1β (96), or the Th2 cytokines, IL-13 and IL-4 (97). Therefore, the infiltrated immune cells are considered as “amplifiers” of airway remodeling (98). Indeed, sustained immune responses are key drivers in exacerbating the development of airway remodeling. For example, cytokines produced by the infiltrated Th2 cells, such as IL-4 and IL-13 enhance subepithelial fibrosis, mucous hyperplasia, and collagen deposition to promote airway remodeling (99–101). Similarly, alveolar macrophages contribute to airway remodeling through the release of matrix metalloproteinases to alter the extracellular matrix (ECM) and airway structure (102). Although the role of Th17 cells in airway remodeling remains controversial, a synergistic effect of DCs together with Th17 cytokines to promote the accumulation of fibrotic matrix components that correlate with TGF-β expression had been observed (103). It is noteworthy that all of above mentioned immune responses are relevant to the suppressive function of Tregs, and therefore, the role of Tregs in airway remodeling has recently been highly appreciated.

Indeed, data collected from animal studies characterized signaling molecules and transcription factors implicated in airway remodeling, which are closely related to Treg function (94, 104, 105). Specifically, transfer of CD4^+^CD25^+^ Tregs at the peak of acute inflammation before the onset of airway remodeling reversed established airway inflammation and prevented the development of airway remodeling (106), supporting a role of Tregs in the prevention of airway remodeling. Similarly, adoptive transfer of tTregs caused a substantial reduction in bronchoalveolar lavage eosinophil composition and suppressed airway remodeling and T cell migration into the lung of STAT6 and RAG2 double knockout mice, confirming the role of Tregs in repressing allergic airway inflammation and remodeling (107). There is also feasible evidence that Th17 responses in chronic allergic airway inflammation abrogate Treg-mediated tolerance, and thereby contributing to airway remodeling (108). More interestingly, adoptive transfer of Tregs into OVA-induced asthmatic mice at the chronic stage selectively reduced the vessel numbers in both peritracheal and peribronchial regions and the lung parenchyma (109), which indicate a potential role of Tregs in the regulation of structural cells such as endothelial cells, smooth muscle cells and mesenchymal cells during the development of airway remodeling in asthmatic settings other than immune cells. Therefore, the exact impact of Tregs on airway remodeling in asthmatic setting is worthy of further investigations.

## The Role of Tregs in Airway Epithelial Repair

Asthmatic patients generally manifest different levels of chronic airway inflammation with airway epithelial damage that occurs even in mild, early and nonfatal asthma (110, 111). Damage and shedding of airway epithelial cells are important pathological features of asthma, and altered epithelium in the airway is more susceptible to injury and apoptosis than those from non-asthmatic individuals (112). Specifically, epithelial cells derived from asthmatic patients collected by bronchial brushing seem to be more hyperreactive and less viable (113), which likely results from inflammatory damage. Furthermore, the airway of asthmatic patients is characterized by the dysregulation of airway epithelial repair, leading to a chronic cycle of wound repair coupled with bronchial remodeling (110).

Except for the immunosuppressive function and capacity to limit the intensity and sustained time of immune responses, Tregs also participate in non-immunological processes such as tissues repair during extensive inflammation. The presence of Tregs has been documented in several non-lymphoid tissues, including lung, skin, placenta, intestinal mucosa, adipose tissue, and atherosclerotic plaques (114). Tregs rapidly accumulate in the acutely injured skeletal muscle of mice. Ablation of Tregs impairs muscle repair due to decreased amphiregulin, an epidermal growth factor family member known to promote healing and tissue regeneration (115). Another study demonstrates that amphiregulin deficiency in Tregs results in severe acute lung damage and a rapid decline in lung function during influenza virus infection. In addition, anti-viral immune responses and suppressive function of Tregs are unaltered, suggesting these two functions are invoked by separable cues (116). Nevertheless, implication of Tregs in airway epithelial repair in the context of asthma has not yet been reported, which is necessary for further investigations.

## Dysregulation of Tregs

Increasing clinical evidence supports that dysregulated Tregs play an important role in the pathogenesis and chronicity of allergic asthma. In patients with asthma and other allergic diseases, the expression of *FOXP3* is reduced as compared to that of healthy controls (117). In atopic children, tTreg maturation is significantly delayed as compared to that of age-matched nonatopic children (118). Additionally, Tregs in patients with allergic asthma exhibit impaired suppressive function compared to those Tregs from healthy controls (11, 119).

Several subsets of dysfunctional Tregs are relevant to allergic asthma. Chemoattractant receptor-homologous molecule expressed on Th2 cells (CRTH2) is one of the functional prostaglandin D2 (PGD2) receptors, and regarded as a potent inducer of type 2 cytokine secretion (48). The allergic asthma patients have more CRTH2^+^ Tregs in the peripheral blood than healthy controls (120). These CRTH2^+^ Tregs produce greater amounts of IL-4 and show less suppressive function than that of CRTH2^−^ Tregs in the PGD2-stimulated cultures (120). Another dysregulated Treg subpopulation is the ILT3 (also known as gp49B or CD85k)-expressing Tregs. Flow cytometry detected a substantially elevated percentage of ILT3^+^ Tregs in mice with massive asthma-like airway pathologies, which promoted the maturation of IRF4^+^PD-L2^+^ DCs to activate Th2 cells (121). Simultaneously, ILT3^+^ Tregs displayed a compromised suppressive function owing to low expression of FOXP3 and Helios (122). In mice, the expression of IL-33 receptor ST2 has been identified in Foxp3^+^ Tregs in the lung (123). In the presence of IL-33, Tregs display upregulated expression of canonical Th2 transcription factor GATA3 and IL-33 receptor ST2 along with enhanced secretion of type 2 cytokines (122). Furthermore, Tregs lose their ability to suppress Teffs once exposed to IL-33 (122). However, *in vitro* studies suggest that ST2^+^ Tregs are highly activated and superior to ST2^-^ Tregs in suppressing CD4^+^ T cell proliferation through IL-10 and TGF-β release (124). Although those *in vitro* data may not mimic the *in vivo* situation, further investigations would be necessary to fully address this question. Pro-inflammatory cytokine-secreting Tregs such as IFN-γ-producing FOXP3^+^ cells, IL-4-producing FOXP3^+^ cells, and IL-17-producing FOXP3^+^ cells are also noticed in asthmatic patients, which are strongly correlated with the severity of asthma and might be insensitive to corticosteroids (125, 126).

## Treg-Based Strategies for Asthma Therapy

It is generally accepted that Tregs are a promising candidate for developing effective therapies to treat immune disorders such as allergic asthma. Current preclinical studies and clinical trials of Treg-based therapies are mainly on the basis of two approaches: one of which is to directly boost Treg number and functionality *in vivo*, and the other is re-administration of purified, *ex vivo* modified, GMP (good manufacturing practice)-compliant Tregs back to patients (127).

Several approaches have been employed to promote the *in vivo* expansion of Tregs or depletion of Teffs, leading to an increased Treg/Teff ratio. These therapies include the administration of IL-2 or IL-2/anti-IL-2 mAb complex, anti-CD3 mAbs, mTOR inhibitors, and dietary or microbe-derived pro-tolerogenic stimulators (127). Although high dose of IL-2 has been used for immunotherapy against metastatic cancer (128), low-dose of IL-2, however, preferentially stimulates Tregs and has shown a great potential of success in Treg-based immunosuppressive strategies against autoimmune and inflammatory diseases (129). Advances in the knowledge of the functional, biophysical and structural characteristics of IL-2 have promoted the generation of IL-2 formulations, such as IL-2/anti-IL-2 mAb complexes (130). Depending on the clone of the anti-IL-2 mAb, IL-2/anti-IL-2 mAb complex exerts differential effect on the expansion of T cell subsets (131). Studies in mice revealed that IL-2/anti-IL-2 mAb complexed with S4B6 clone induces the preferential proliferation of CD8^+^ T cells, while the IL-2/JES6-1 complex preferentially induces the expansion of Tregs by blocking the interaction of IL-2 with IL-2Rβ (CD122) and IL-2Rγ (CD132), and promoting the interaction of IL-2 with IL-2Rα (CD25) (131, 132). Particularly, the IL-2/JES6-1 complexes have already manifested exciting results in terms of suppressing organ transplant rejection (133), autoimmune and inflammatory diseases in mice such as type 1 diabetes (134), dextran sodium sulfate-induced colitis (132), experimental myasthenia (135), collagen-induced arthritis (136), experimental autoimmune encephalomyelitis (133), and allergic airway disease (137). In the model of established airway allergy, treatment with IL-2/JES6-1 complex dampens eosinophilia and airway inflammation, and inhibits the production of eotaxin-1 and IL-5 (137). Mucus production, AHR to methacholine, and parenchymal tissue inflammation are also dramatically reduced following IL-2/anti-IL-2 mAb complex administration, which is dependent on Treg-derived IL-10 (137). Interestingly, administration of IL-2/JES6-1 complex also improves some manifestations of metabolic diseases, such as obesity related chronic inflammation and insulin resistance, which are characterized by the inflammatory infiltration of immune cells in the adipose tissues that are amenable to Treg modulation (138).

In neoplastic diseases, adoptive cell therapies (ACT) use T cells engineered to express either Ag-specific TCRs or chimeric Ag receptors (CARs) targeting specific tumor antigens to selectively eliminate target cells, which have been approved for the treatment of acute lymphoblastic leukemia and advanced lymphomas (127). In addition to killing cancerous cells, ACT can also be used to regain appropriate Treg function in the inflammatory context. Polyclonal expansion of Tregs *via* TCR represents the initial strategy for ACT. Unlike other type of Tregs, antigen-specific Tregs are more potent in controlling local inflammation and inhibiting T cell priming in secondary lymphoid tissues (139). More recently, a number of publications demonstrate the utility of CARs in Tregs (140). In this case, Tregs are reinfused after engineering with chimeric TCR of different types. CAR-Tregs have several characteristics versus TCR-Tregs: (1) non-MHC-restriction and less dependent on IL-2; (2) the hinge region provides flexibility, which enables CARs binding to antigen in various orientations; (3) higher antigen affinity than TCRs; and (4) more precise control of the type of antigen-stimulated response (141).

Current preclinical studies and clinical trials for Treg ACTs in inflammatory disorders have indicated the efficacy and technical feasibility of these methods (142, 143). In experimentally induced allergic asthma, CAR-redirected Tregs suppressed allergic airway inflammation, prevented excessive pulmonary mucus production, and attenuated the increase of allergen-specific IgE and Th2 cytokine levels (144). Over the past few decades, autoimmune involvement in the pathogenesis of asthma has been proposed due to the presence of circulating autoantibodies against diverse self-antigens/structures (145). Some patients with severe asthma have autoantibodies against eosinophil peroxidase (EPX) and autologous cellular components in the sputum, which may necessitate an increase for the maintenance of corticosteroids (146). These findings raise the potential of utilizing CAR-Treg ACT in severe and therapy-refractory asthmatics. However, many important issues such as managing the stability and plasticity of Tregs, directing their homing to the desired sites, and safety concerns are still waiting to be worked out.

## Conclusion Remarks

Allergic asthma involves complex innate and adaptive immune responses to environmental allergens, resulting in airway inflammation predominately mediated by Th2-type cells and allergen-specific IgE (147). Both human and animal studies show that Tregs are essential for the maintenance of self-tolerance and immune homeostasis, and therefore, Tregs defects are observed in asthmatic individuals as compared to healthy controls in terms of their functionality. These discoveries promoted the development of technologies with Treg-based therapies, such as Treg expansion and CAR-Tregs, which may represent a viable approach for curative therapy of allergic diseases. Despite the current achievements, some critical issues, such as how to improve the safety of Tregs, increase the stability of Tregs, and direct their homing to the desired sites, are yet to be elucidated. As a result, additional in-depth studies are necessary to improve current therapeutic approaches against asthma in clinical settings.

## Author Contributions

JZ, YZ, and LC wrote the manuscript and prepared the figure. QX collected and analyzed the information. YW, MX, and XL reviewed the manuscript. JPZ and C-YW supervised the conception and writing of the manuscript. All authors contributed to the article and approved the submitted version.

## Funding

Our study was supported by the National Natural Science Foundation of China (82130023, 81920108009, 82100892, 82070808, 81873656, 82100823, 82100931, 91749207, 81770823 and 81800068), Department of Science and Technology of Hubei Province Program project (2020DCD014), the Postdoctoral Science Foundation of China (54000-0106540081 and 54000-0106540080), Hubei Health Committee Program (WJ2021ZH0002), the Integrated Innovative Team for Major Human Diseases Program of Tongji Medical College, Huazhong University of Science and Technology, and the Innovative Funding for Translational Research from Tongji Hospital.

## Conflict of Interest

The authors declare that the research was conducted in the absence of any commercial or financial relationships that could be construed as a potential conflict of interest.

## Publisher’s Note

All claims expressed in this article are solely those of the authors and do not necessarily represent those of their affiliated organizations, or those of the publisher, the editors and the reviewers. Any product that may be evaluated in this article, or claim that may be made by its manufacturer, is not guaranteed or endorsed by the publisher.
